# Impaired relaxation despite upregulated calcium-handling protein atrial myocardium from type 2 diabetic patients with preserved ejection fraction

**DOI:** 10.1186/1475-2840-13-72

**Published:** 2014-04-05

**Authors:** Regis R Lamberts, Shivanjali J Lingam, Heng-Yu Wang, Ilse AE Bollen, Gillian Hughes, Ivor F Galvin, Richard W Bunton, Andrew Bahn, Rajesh Katare, J Chris Baldi, Michael JA Williams, Pankaj Saxena, Sean Coffey, Peter P Jones

**Affiliations:** 1 Department of Physiology - HeartOtago, Otago School of Medical Sciences, University of Otago, Dunedin, New Zealand; 2 Department of Cardiothoracic Surgery, Dunedin School of Medicine, Dunedin Hospital, Dunedin, New Zealand; 3 Department of Medicine – HeartOtago, Dunedin School of Medicine, Dunedin Hospital, Dunedin, New Zealand

**Keywords:** Diastolic dysfunction, Human myocardium, Type 2 diabetes, Relaxation, Contraction, Adrenergic regulation, Calcium signaling, Myocardial fibrosis

## Abstract

**Background:**

Diastolic dysfunction is a key factor in the development and pathology of cardiac dysfunction in diabetes, however the exact underlying mechanism remains unknown, especially in humans. We aimed to measure contraction, relaxation, expression of calcium-handling proteins and fibrosis in myocardium of diabetic patients with preserved systolic function.

**Methods:**

Right atrial appendages from patients with type 2 diabetes mellitus (DM, n = 20) and non-diabetic patients (non-DM, n = 36), all with preserved ejection fraction and undergoing coronary artery bypass grafting (CABG), were collected. From appendages, small cardiac muscles, trabeculae, were isolated to measure basal and β-adrenergic stimulated myocardial function. Expression levels of calcium-handling proteins, sarcoplasmic reticulum Ca^2+^ ATPase (SERCA2a) and phospholamban (PLB), and of β_1_-adrenoreceptors were determined in tissue samples by Western blot. Collagen deposition was determined by picro-sirius red staining.

**Results:**

In trabeculae from diabetic samples, contractile function was preserved, but relaxation was prolonged (Tau: 74 ± 13 ms vs. 93 ± 16 ms, non-DM vs. DM, p = 0.03). The expression of SERCA2a was increased in diabetic myocardial tissue (0.75 ± 0.09 vs. 1.23 ± 0.15, non-DM vs. DM, p = 0.007), whereas its endogenous inhibitor PLB was reduced (2.21 ± 0.45 vs. 0.42 ± 0.11, non-DM vs. DM, p = 0.01). Collagen deposition was increased in diabetic samples. Moreover, trabeculae from diabetic patients were unresponsive to β-adrenergic stimulation, despite no change in β_1_-adrenoreceptor expression levels.

**Conclusions:**

Human type 2 diabetic atrial myocardium showed increased fibrosis without systolic dysfunction but with impaired relaxation, especially during β-adrenergic challenge. Interestingly, changes in calcium-handling protein expression suggests accelerated active calcium re-uptake, thus improved relaxation, indicating a compensatory calcium-handling mechanism in diabetes in an attempt to maintain diastolic function at rest despite impaired relaxation in the diabetic fibrotic atrial myocardium. Our study addresses important aspects of the underlying mechanisms of diabetes-associated diastolic dysfunction, which is crucial to developing new therapeutic treatments.

## Background

Impaired cardiac function during diastole is one of the early manifestations of cardiac dysfunction in type 2 diabetes [1], and it makes type 2 diabetic patients prone to developing clinical features of heart failure, with both preserved and reduced ejection fraction (EF) [2,3]. Diastolic function deteriorates progressively over time [4], but the exact mechanisms underlying this clinically important increased stiffness and/or impaired relaxation, especially in diabetes, remain unclear. In contrast to systolic dysfunction, treatments for diastolic dysfunction are limited, which is likely due to this lack of knowledge regarding the pathophysiology of diastolic dysfunction [5].

The diastolic properties of the heart are determined by factors influencing both the passive compliance and the active processes of myocardial relaxation [6]. Thus far, studies using animals with type 1 diabetes [7] and diabetic patients with reduced EF [8] have found increased collagen deposition, which stiffens the extracellular myocardial matrix. More recently, the slow/incomplete removal of Ca^2+^ from the cytosol during the diastolic period, predominantly governed by the activity of the important sarcoplasmic reticulum Ca^2+^ ATPase (SERCA2a) under direct control of its endogenous inhibitor phospholamban (PLB) [9], has been shown to contribute to the impaired myocardial relaxation in early diastolic dysfunction [10]. However, the underlying changes in these important calcium-handling proteins are yet to be examined in human diabetic myocardium with preserved EF. Moreover, as impaired diastolic function limits cardiac performance during exercise [11] and leads to an intolerance to tachycardia [12], it is also crucial to determine how relaxation is challenged by β-adrenergic stimulation, one of the most important extrinsic physiological stimulus of the myocardium.

Therefore we aimed to assess the impact of diabetes on the diastolic function of the atrial myocardium from patients with preserved EF and determine alterations in the calcium-handling proteins specifically linked to myocardial relaxation. To this end we combined active force and relaxation measurements in isolated right atrial cardiac muscles from non-diabetic and diabetic patients with preserved EF and coronary artery disease (CAD), undergoing coronary artery bypass graft (CABG) surgery. Expression levels of calcium-handling proteins and β_1_-adrenoreceptors were determined via protein analysis and atrial myocardial fibrosis by measuring collagen deposition.

Our study shows for the first time that in human atrial myocardium from diabetic patients with preserved EF and CAD, the force generation is preserved, whereas the relaxation is impaired, and the right atrial cardiac muscles were unresponsive to β-adrenergic stimulation. The impairment in relaxation is associated with an unexpected increase in SERCA2a/PLB protein ratio in the diabetic myocardium suggesting accelerated relaxation. This may indicate a compensatory mechanism in an attempt to maintain diastolic function at rest in the fibrotic diabetic atrial myocardium.

## Methods

### Myocardial samples

The study was approved by the local Human Ethics Committee (Approval number: LRS12-01-001) and informed consent was obtained. Human right atrial appendages (RAAs) were acquired from patients with type 2 diabetes mellitus (DM, n = 20) and non-diabetic patients (non-DM, n = 36) with CAD who underwent CABG surgery. Non-diabetic patients were classified as having a fasting plasma glucose ≤ 6 mmol/L or an HbA1_c_ score ≤ 6.5% (48 mmol/mol). DM patients were selected based on their clinical diagnosis of type 2 diabetes with duration of more than 1 year. CAD was of such severity as to meet the indications for CABG, which in the majority of cases was triple vessel or high-grade double vessel disease (number of grafts per patient 3.7 ± 0.2 vs. 3.5 ± 0.3, non-DM vs. DM, p = 0.68). Patients requiring emergency CABG, patients with on-going myocardial ischemia prior to CABG and those undergoing concomitant cardiac surgical procedures in addition to CABG were excluded.

### Echocardiography

All patients had a clinically indicated pre-operative transthoracic echocardiogram, using a Vivid S6, 7, or E9 ultrasound system (GE Vingmed Ultrasound, Horten, Norway). Images were obtained by professional echosonographers, and reviewed offline by a European Association of Cardiovascular Imaging accredited cardiologist. Left ventricular (LV) and left atrial (LA) chamber volumes were derived from a biplane method of discs using the apical 4-chamber and 2-chamber views [13]. Cardiac output measurements were calculated applying the heart rate recorded during the apical 4-chamber view. Ejection fraction (EF) was graded according to the European Association of Echocardiography/American Society of Echocardiography recommendations as preserved (> 50%) [14]. LV mass was calculated according Devereux et al. [15], whereas LV volume/mass ratio was calculated at end-systole (LVESV/LV mass) [16].

### Tissue acquisition and preservation

In all patients, RAAs were removed under normothermic conditions before cross clamping for cardiopulmonary bypass. Immediately after removal, all specimens were placed in a sealed vial containing a modified, low Ca^2+^ (0.5 mM) Krebs-Henseleit buffer ((mM): 118.5 NaCl, 4.5 KCl, 1.4 CaCl_2_, 0.3 NaH_2_PO4, 1.0 MgCl_2_6H_2_O, 25 NaHCO_3_ and 11 glucose) with 6.25 mM 2,3-butanedione monoxime (BDM) previously well oxygenated with carbogen (95%O_2_–5%CO_2_). Within 5–10 minutes after removal, the appendages were either dissected into several pieces, flash-frozen and stored at 80°C or fixed in 4% (v/v) formaldehyde solution and stored at -20°C. In a subset of the study, half of the RAA tissue was used immediately to conduct the functional force measurements.

### Functional force measurements

From myocardial samples, tiny cardiac muscles (trabeculae) were freshly dissected, transferred to an experimental bath and attached between a force transducer and a micromanipulator. The basal and length dependency of force development were measured in the isolated trabeculae, as previously described [17]. The muscles were constantly superfused with a modified oxygenated Krebs–Henseleit solution, as indicated above but without BDM and at 1.4 mM Ca^2+^, kept at 40°C and continuously stimulated at 60 bpm (1 Hz). After mounting, the preparations were stimulated for 1 h to allow equilibration. To impose similar stretch levels the muscles in both groups were stretched to the length (L_max_) at which isometric developed force was maximal. After determination of basal force developed (F_dev_), length dependency was tested by changing muscle length from 80% to 100% L_max_ with 5% incrementing steps. Hereafter the response to β-adrenoceptor stimulation on force development was tested with incrementing doses of dobutamine (10^-7^ to 10^-5^ M) given at 6 minutes interval.

### Tissue lysate preparation and protein analysis

Total myocardial sample lysates were prepared by manual grinding and followed by suspension in 1.5-fold (w/v) sodium dodecyl sulphate (SDS) lysis buffer (3% (w/v) SDS, 50 mM Tris–HCl (pH 7.5) and protease inhibitor mix composed of 1 mM benzamidine, 2 μg/mL leupeptin, 2 μg/mL pepstatin A, 2 μg/mL aprotinin and 0.5 mM phenylmethylsulfonyl fluoride). The homogenate was incubated on ice for 1 hour, at room temperature for 45 minutes and then centrifuged at 21,000 g for 30 minutes at 4°C where after supernatant was collected. Protein concentrations were determined using the DC protein determination kit (BioRAD) according to manufacturer’s protocol and samples volumes were adjusted for loading. Unless otherwise noted samples were further solubilized in 2× Laemmli sample buffer (65.8 mM Tris–HCl, pH 6.8, 2.1% SDS, 26.3% (w/v) glycerol, 0.01% bromophenol blue) containing 5% β-mercaptoethanol (v/v). Ryanodine receptor (RyR2), SERCA2a, PLB and β_1_-adrenoreceptor were detected from 75, 60, 50 or 25 μg of lysate, respectively, and glyceraldehyde 3-phosphate dehydrogenase (GAPDH) or β-actin was also identified in each loading condition. Following transfer onto nitrocellulose the membranes were blocked for 1 hour with phosphate buffered saline (PBS, pH 7.4), containing 0.5% Tween 20 and 5% (w/v) skimmed milk. For β_1_-adrenoceptor and β-actin proteins were transferred onto Polyvinylidene fluoride (PVDF) membranes, blocked for 1 hour with Tris buffered saline (TBS, pH 7.4). The blots were probed with specific antibodies against RyR2, SERCA2a, PLB, GAPDH (*Badrilla Ltd*), β_1_-adrenoreceptor (*Novus Biologicals*) or β-actin (*Sapphire Bioscience Pty.Ltd*) with corresponding peroxidase conjugated secondary antibodies (*Sapphire Bioscience Pty.Ltd)*. All proteins were detected using an enhanced chemiluminescence kit. Protein band densities were normalized to GAPDH or β-actin as an internal control and are expressed as protein to GAPDH or β-actin ratio. For β_1_–adrenoceptor (AR) blots, β-actin was used as an internal control because GADP was to close to the β_1_-AR bands to be separated accurately. Although some studies suggested an impact of diabetes on the *gene* expression of GAPDH [18], others [19] and ours did not observe any changes in *protein* expression of GAPDH or of β-actin (both p > 0.05) between non-DM and DM samples.

### Fibrosis determination

Formalin fixed, optical cutting temperature embedded myocardial samples tissue sections of 8 μm thickness were washed with distilled water and PBS, and subsequently exposed to a picro-sirius red solution (0.001% (w/v) sirius red in saturated aqueous picric acid) for 1 hour. Tissue sections were then washed in acidified H_2_O (0.5% (v/v) acetic acid in H_2_O) for 10 minutes. Slices were dehydrogenated in an ascending ethanol sequence (85%, 95% and 100% (v/v) in H_2_O) and washed in xylene. Finally, slices were embedded in a distrene-plasticiser-xylene mounting medium. Images obtained by light microscopy were digitized and analyzed using ImageJ v1.46 software to quantify the percentage of collagen content (fibrosis), as previously described [20]. Briefly, the number of red pixels above a set threshold (similar for all images) was calculated from the total pixel area of interest. The area of cardiomyocytes and collagen, excluding the lumen (i.e. empty spaces), determined the total area of interest. The mean percentage of collagen deposition from 21 randomly selected images was calculated to represent the level of collagen of one RAA.

### Statistical Analysis

Differences among groups were compared either using a t-test, chi-square or a two-way ANOVA followed by a Bonferroni post-hoc test as indicated in the legends of the tables and Figures. A p-value of < 0.05 was considered statistically significant. Data are expressed as mean ± SEM.

## Results

### Clinical characteristics

Diabetes was evident from the high fasting levels of glycated hemoglobin (HbA_1c_) (58 ± 4 mmol/mol in DM group, normal below 48 mmol/mol) and fasting plasma blood glucose levels (10.0 ± 1.0 mmol/L in DM group, normal below 6.1 mmol/L) (Table 1). All patients had preserved EF (> 50%), and no differences in blood pressures, heart rate, cardiac dimensions, LV mass or LV volume/mass ratio were observed between both groups, suggesting normal systolic function and no additional structural remodeling in DM patients with CAD compared to the non-DM patients with CAD. Although almost all patients were previously diagnosed with hypertension, the incidence was not different between non-DM and DM patients, whereas non-hypertensive blood pressure values indicate the effectiveness of the blood pressure lowering treatment. In contrast, diastolic function was reduced in DM compared to non-DM patients (Table 1) as indicated by a significantly slower early filling time (prolonged early deceleration time, p < 0.05), by a significantly elevated marker of ventricular filling pressures (increased E/e’, p < 0.05) and by the tendency to slower early and late filling velocity (increased E and A, p = 0.05 and p = 0.08, respectively).

**Table 1:** Patient characteristics

***Clinical characteristics***	**Non-DM (n = 36)**	**DM (n = 20)**	**P value**
Age (years)	67 ± 1	68 ± 2	0.58
Gender (male/female)	28/8	14/6	0.52
BMI (kg/m^2^)	28.2 ± 0.9	33.8 ± 1.6	0.0015*
Blood glucose (mmol/L)	5.9 ± 0.3	10.0 ± 1.0	<0.0001*
HbA1_c_ (%) (mmol/mol)	5.8 ± 0.3 (40 ± 1)	7.5 ± 1.2 (58 ± 4)	0.0011*
Diabetes duration (years)	-	11.6 ± 2.7	
Diagnosed Hypertension	29/36	18/20	0.47
***Echocardiography***			
Systolic BP (mmHg)	129 ± 3	133 ± 4	0.49
Diastolic BP (mmHg)	73 ± 2	70 ± 2	0.40
Heart rate (bpm)	64 ± 1	69 ± 2	0.07
LVEDV (mL)	104 ± 3	95 ± 5	0.20
LVESV (mL)	43 ± 2	39 ± 4	0.43
SV (mL)	61 ± 2	56 ± 3	0.17
Cardiac Output (L/min)	3.8 ± 0.1	4.0 ± 0.1	0.47
Ejection Fraction (%)	60 ± 1	60 ± 2	0.86
IVSd (cm)	1.14 ± 0.04	1.26 ± 0.06	0.08
LVPWd (cm)	1.05 ± 0.03	1.15 ± 0.06	0.15
LVIDd (cm)	4.76 ± 0.09	4.72 ± 0.15	0.81
LVIDs (cm)	3.04 ± 0.10	3.04 ± 0.14	0.99
LV mass (g)	193 ± 11	217 ± 18	0.22
LV volume/mass (ml/g)	0.22 ± 0.03	0.19 ± 0.03	0.23
E (m/sec)	0.68 ± 0.03	0.79 ± 0.05	0.05
A (m/sec)	0.69 ± 0.03	0.80 ± 0.05	0.08
E/A	1.05 ± 0.08	0.99 ± 0.08	0.63
e' (m/sec)	0.069 ± 0.003	0.067 ± 0.004	0.63
E/e'	10.5 ± 0.8	12.6 ± 1.1	0.01*
Decel (msec)	226 ± 7	259 ± 13	0.02*
LA-2D (cm)	4.0 ± 0.1	4.1 ± 0.2	0.55
***Medication***			
Statins	35/36 (97%)	20/20 (100%)	0.45
Beta-blockers	31/36 (86%)	15/20 (75%)	0.30
ACE inhibitors/AT blockers	19/36 (53%)	19/20 (95%)	0.0012#
Calcium Channel blocker	7/36 (19%)	8/20 (40%)	0.10
Metformin	0/36 (0%)	14/20 (70%)	
Insulin	0/36 (0%)	6/20 (30%)	

BMI – body mass index; BP – blood pressure; HbA1_c_ – glycated haemoglobin; LVEDV: left ventricular end diastolic volume; LVESV: left ventricular end systolic volume; SV: stroke volume; IVSd: interventricular septal diameter diastole; LVPWd: left ventricular posterior wall thickness end-diastole; LVIDd: left ventricular internal diameter diastole; LVIDs: left ventricular internal diameter systole; LV volume/mass: LVESV / LV mass; E: early ventricular filling velocity; e’: early ventricular myocardium relaxation velocity; A: late ventricular filling velocity; Decel: early ventricular deceleration time; LA-2D: left atrial 2-dimensional diameter. Data are mean ± SEM; For values: * = p < 0.05 vs. non-diabetic, unpaired t-test; For ratios: # = p < 0.05 vs. non-diabetic, Chi-square test.

### Human atrial myocardial function

**Figure 1:**
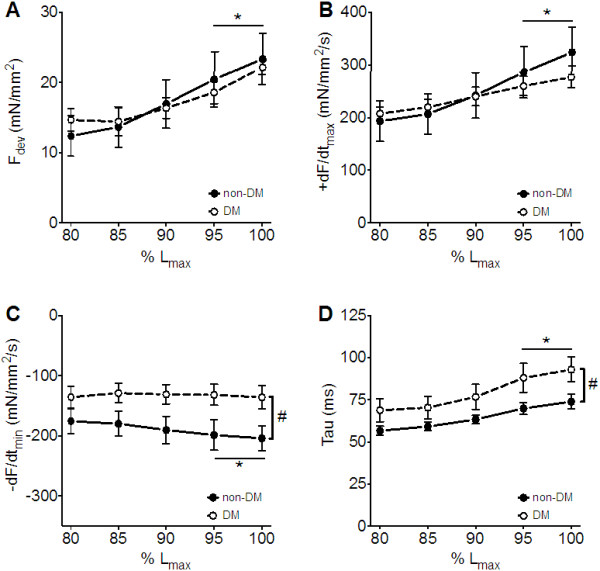
**Contraction and relaxation during force-length relationship in human right atrial cardiac muscles.** Preserved force-length relationship from 80 to 100% L_max_ in isolated isometric contracting right atrial cardiac muscles from non-diabetic (non-DM, closed circles, n = 8) and type 2 diabetic (DM, open circles, n = 6) patients. The contractile parameters, developed force (F_dev_, **A**) and maximum rate of contraction (+dF/dt_max_, **B**) were not different between both groups. However, both relaxation parameters, minimum rate of relaxation (-dF/dt_min_, **C**) and time constant of relaxation (Tau, **D**) were impaired in muscles from type 2 diabetic patients. Overall this indicates preserved contraction but impaired early and late relaxation in human diabetic right atrial myocardium with preserved LV ejection fraction. Two-way ANOVA: length effect: * = p < 0.05 vs. 80% L_max_ for both non-DM and DM group; Group effect: # = p < 0.05 vs. non-DM. Data are mean ± SEM*.*

The dimensions of right atrial trabeculae dissected were not different between groups (Table 2) and were well below the range that oxygen diffusion impact the core of the trabeculae [21]. Under resting conditions (1 Hz, L_max_), the contractile parameters (F_dev_, +dF/dt_max_) were not different between muscles from non-DM and DM patients, however the relaxation parameters, minimum rate of relaxation (-dF/dt_min_), 50% relaxation time (RT50%) and time constant of relaxation (Tau), were significantly prolonged in DM group (Table 2). Figure 1 shows that the force-length relationship, one of the most important intrinsic functional regulatory mechanisms of the heart (the Frank-Starling mechanism), was preserved in both cohorts, as indicated by increasing contractile parameters (F_dev_ and + dF/dt_max_) with incrementing length (Figure 1A and B). In contrast to the lack of difference in contractile parameters between cardiac muscle from non-DM and DM patients, the diabetic muscles showed a significant decrease in -dF/dt_max_ (Figure 1C) and a prolonged Tau (Figure 1D) at all lengths indicating impaired relaxation. Together these data indicate preserved contraction but impaired relaxation in cardiac muscles from DM patients with preserved EF.

**Table 2:** Basal characteristics and functional parameters of human cardiac muscle

***Muscle characteristics***	**Non-DM (n = 8)**	**DM (n = 6)**	**P value**
CSA (mm^2^)	0.046 ± 0.036	0.051 ± 0.043	0.84
L_max_ (mm)	1.73 ± 0.58	1.34 ± 0.15	0.18
***Contractile parameters***			
F_dev_ (mN/mm^2^)	23.7 ± 3.6	23.1 ± 1.1	0.91
+dF/dt_max_ (mN/mm^2^/s)	298 ± 37	277 ± 21	0.70
TtPF (ms)	145 ± 16	139 ± 7	0.77
***Relaxation parameters***			
-dF/dt_min_ (mN/mm^2^/s)	-204 ± 20	-130 ± 16	0.02*
RT50% (ms)	166 ± 9	209 ± 6	0.01*
Tau (ms)	74 ± 4	93 ± 7	0.03*

CSA - cross sectional area; L_max_- muscle length at maximal force development; F_dev_ - developed force; +dF/dt_max_ - maximum rate of contraction; TtPF - time to peak force; **-**dF/dt_min_ - minimum rate of relaxation; RT50% - 50% relaxation time; Tau - time constant of relaxation. Data are mean ± SEM; * = p < 0.05 vs. non-diabetic, unpaired t-test.

### Calcium-handling proteins expression levels

**Figure 2:**
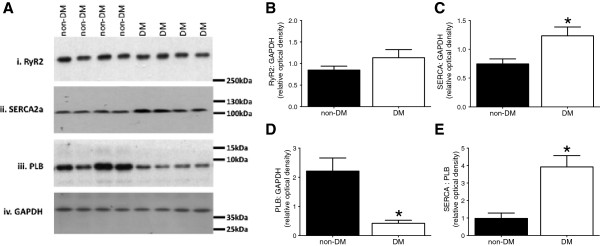
**Expression of calcium-handling proteins. A**. Membranes containing human right atrial cardiac tissue samples from non-diabetic (non-DM) and diabetic (DM) patients were probed for RyR2, SERCA2a, PLB, and GAPDH. RyR2 was detected above 250 kDa, SERCA2a between 100 kDa and 130 kDa, PLB below 10 kDa and for all samples GAPDH above 35 kDa as loading control. Western blot analysis showed similar protein expression for **B**. RyR2, whereas **C**. SERCA2a expression was increased and **D**. the expression of PLB was more than five times lower in the cardiac tissue of diabetic patients. **E**. Consequently the SERCA2a:PLB ratio showed a four times increase in the diabetic group compared to non-diabetic group. Data are means ± SEM, obtained from four replica experiments using non-DM (n = 16) and DM (n = 8) patient samples, except non-DM for RyR2 from n = 12; * = p < 0.05 vs. non-DM, unpaired t-test.

To evaluate whether changes in expression of calcium-handling proteins might contribute to the observed impaired relaxation in diabetes, the expression of RyR2, SERCA2a and PLB were determined in the right atrial samples (Figure 2). In line with the preserved contractile function observed in DM muscles, the expression of the SR Ca^2+^ release protein RyR2 was not different between both groups of patients (0.85 ± 0.09 vs. 1.14 ± 0.19, non-DM vs. DM, p = 0.14; Figure 2B). However surprisingly, despite the impaired relaxation, the expression of SERCA2a, responsible for SR Ca^2+^ re-uptake was increased in DM patients (0.75 ± 0.09 vs. 1.23 ± 0.15, non-DM vs. DM, p = 0.007; Figure 2C). Moreover, the expression of PLB, the endogenous inhibitor of SERCA2a, was decreased in the cardiac tissue of DM patients (2.21 ± 0.45 vs. 0.42 ± 0.11, non-DM vs. DM, p = 0.01; Figure 2D). Consequently the SERCA2a:PLB ratio, which correlates with SERCA2a activity, showed an increase in DM group (0.98 ± 0.31 vs. 3.92 ± 0.65, non-DM vs. DM, p = 0.0001; Figure 2E), suggesting significantly enhanced SR Ca^2+^ re-uptake.

### Human atrial myocardial functional response to β-adrenergic stimulation

**Figure 3:**
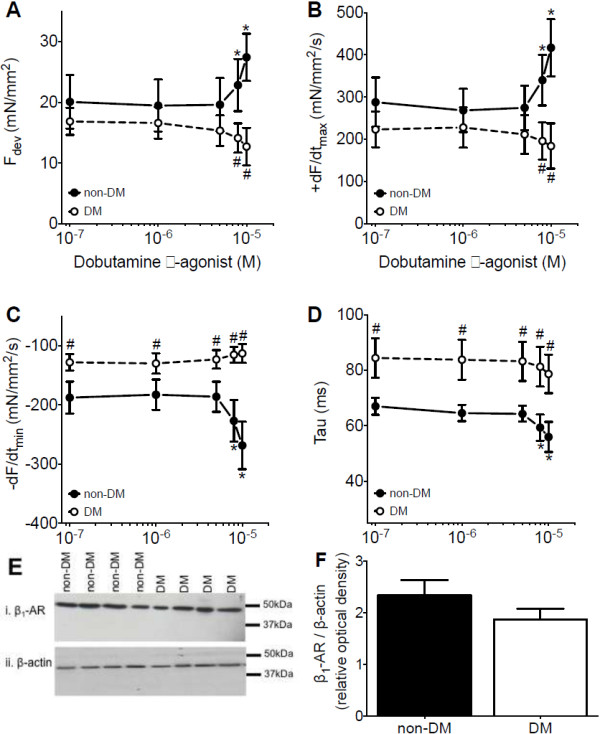
**Myocardial function during β-adrenergic stimulation.** In response to incrementing doses of β-adrenergic agonist dobutamine (10^-7^ to 10^-5^ M) right atrial cardiac muscles from non-diabetic patients (non-DM, closed circles, n = 8) showed an increase in amplitude of contraction (F_dev_, **A**), and speed of contraction (+dF/dt_max_, **B**); and a faster speed of early relaxation (-dF/dt_min_, **C**) and late relaxation (Tau, **D**). Strikingly, both the increase in the contractile parameters (F_dev_ and + dF/dt_max_) and the hastening of the relaxation parameters (-dF/dt_min_ and Tau) were absent in the cardiac muscles from the diabetic patients (DM, open circles, n = 6). **E**. Western blotting was performed with human cardiac tissue samples from non-diabetic (non-DM) and diabetic (DM) patients probed for β_1_–adrenoceptor (β_1_-AR) and β-actin. In representative blot β_1_–AR was detected just below 50 kDa and β-actin above 37 kDa as loading control. **F**. Western blot analysis showed similar protein expression for β_1_–adrenoceptor. For all functional parameters two-way ANOVA Bonferroni’s posthoc: dose effect, * = p < 0.05 and DM effect, # = p < 0.05). For protein analysis data obtained from four replica experiments, non-DM (n = 10) and DM (n = 9) patients, unpaired t-test. Data are mean ± SEM.

As impaired diastolic function limits cardiac performance during exercise [11], we also evaluated whether the impaired relaxation observed in the diabetic right atrial myocardium might be further compromised during a physiological challenge. Therefore, the human right atrial cardiac muscles were also exposed to β-adrenergic stimulation using incrementing doses of dobutamine (10^-7^ to 10^-5^ M). As expected, this resulted in an increase in the amplitude of contraction (F_dev_, Figure 3A), speed of contraction (+dF/dt_max_, Figure 3B), speed of early relaxation (-dF/dt_min_, Figure 3C) and of late relaxation (Tau, Figure 3D) in muscles from non-DM patients. Strikingly, the β-adrenergic stimulation-induced increase in contractility (F_dev_ and + dF/dt_max_, Figure 3A and B) and acceleration of relaxation (-dF/dt_min_ and Tau, Figure 3C and D) were absent in muscles from DM patients. Using western blot analysis we assessed whether the impaired inotropic and lusitropic β-adrenergic responsiveness in diabetic myocardium was related to reduced β_1_-adrenoreceptor content. However, as shown in Figure 3F the expression levels of the β_1_-adrenoreceptor were not different between cardiac tissue samples from the non-DM and DM patients (2.3 ± 0.3 vs. 1.9 ± 0.2 non-DM vs. DM, p = 0.22).

### Myocardial fibrosis

**Figure 4:**
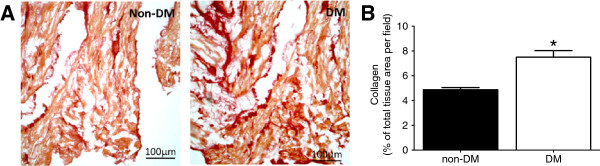
**Collagen deposition. A**. Representative images showing collagen density of picro-sirius red stained right atrial myocardial tissue samples from non-diabetic (non-DM, n = 6) and diabetic (DM, n = 7) patients; yellow/brown staining represents myocardial fibers and red staining represents collagen fibers. **B**. Calculated collagen deposition was increased in DM group compared to non-DM group; collagen deposition reported as % of collagen in total tissue area/field from 21 fields/sample. Data are mean ± SEM; * = p < 0.05 vs. non-DM, unpaired t-test.

In addition to impaired relaxation, changes in myocardial stiffness could also lead to diastolic dysfunction, therefore we next assessed whether increased fibrosis exists in the diabetic right atrial myocardium. Collagen deposition was determined in cardiac tissue samples using picro-sirius red, which selectively stains collagen red. A 60% increase in collagen deposition was observed in DM patients (4.9 ± 0.2% vs. 7.5 ± 0.5%, non-DM vs. DM, p = 0.003; Figure 4), indicating increased myocardial fibrosis in DM patients.

## Discussion

Our findings show that in human right atrial myocardium from type 2 diabetic patients, with CAD and preserved EF, the contractile state is preserved, whereas relaxation is impaired, despite an increase in SERCA2a:PLB expression ratio. This is associated with an increased level of atrial myocardial fibrosis. The increase in SERCA2a:PLB ratio may suggest a compensatory mechanism in an attempt to maintain diastolic function at rest despite impaired relaxation in the fibrotic diabetic atrial myocardium. We also show for the first time that human right atrial cardiac muscles from patients with type 2 diabetes and preserved EF are unresponsive to β-adrenergic stimulation without a change in expression level of the β_1_-adrenoreceptors, suggesting impaired downstream β-adrenergic modulation. Our study provides novel information on the underlying pathology of diastolic dysfunction in the diabetic myocardium, strengthening the knowledge base for the development of therapeutics, which are currently very limited for diastolic dysfunction [5,22].

### Impaired relaxation in human right atrial diabetic myocardium at rest

Our *in vivo* echocardiography data and our *ex vivo* cardiac muscle data confirmed both preserved contractile function with no development of structural cardiac hypertrophy in diabetic patients with preserved EF. Echocardiography of the left ventricle also revealed a slower early filling time (prolonged deceleration time) and higher filling pressures during early filling (increased E/e’) with a tendency to slower early and late filling velocities (E and A, respectively), which in the isolated right atrial muscles were accompanied by prolongations of early and late relaxation (decreased -dF/dt_min_ and prolonged Tau). Together these data indicate reduced diastolic function due to impaired relaxation and increased myocardial stiffness of the heart in the diabetic population with preserved EF. This is consistent with previous echocardiography findings in type 1 and type 2 diabetic patients [2,23] and with functional myocardial studies in type 1 [24] and type 2 diabetic animal models [25], however the myocardial diastolic dysfunction observed in animal models was always accompanied by systolic dysfunction. On the contrary, Reuter *et al.* have reported no difference in the rate of relaxation or the contractile function of right atrial cardiac muscles from type 2 diabetic patients undergoing CABG [26]. However, the contractile and relaxation measurements reported in that study were much lower in magnitude when compared to values reported in other studies using human right atrial cardiac muscles [27,28] and our study (Table 2). This may be related to tissue degradation in their samples due to prolonged loading with a fluorescence indicator, while our experimental measurements were obtained within 90 minutes after dissection.

The heart has the ability to increase its cardiac efficiency via intrinsic means, such as volume [29]. Several studies reported that the Frank-Starling mechanism is maintained in patients with reduced EF [28,30]. More recently, patients with preserved EF showed a preserved static (intrinsic to the heart) Frank-Starling mechanism [31], whereas the beat-to-beat dynamic (including the heart-arterial interaction) Frank-Starling mechanism was impaired in these patients [32]. By increasing the length of our isolated cardiac muscles we mimicked the myocardial stretch and recorded a similar increase in force development in both our non-DM and DM groups. This indicates a preserved Frank-Starling mechanism in diabetic patients with preserved EF, and supports the concept that arterial stiffening might be more important for the observed impaired dynamic Frank-Starling mechanism during preserved EF than myocardial stiffening [32].

### Impaired relaxation during physiological challenge

Although contractile function at rest was not altered by diabetes, the relaxation of the right atrial diabetic muscles was impaired over the entire range of myocardial stretch, and both the inotropic and lusitropic responses to β-adrenergic stimulation were completely absent in cardiac muscles from DM patients. A reduced but still responsive myocardium to β-adrenergic stimulation has been observed in many diabetic animal models [33,34] and has been related to reduced expression levels of β_1_-adrenoreceptors [35]. Surprisingly, cardiac muscles from our cohort of diabetic patients failed to respond to the β-adrenergic stimulation, even at very high (10^-5^ M) concentrations, which would indicate a lack of contractile reserve even in these patients with normal resting contractile function. In addition, our new data emphasizes that caution should be taken with translating animal data directly to the human situation. It would be easy to attribute this to the use of β-adrenoceptor blocking agents, however almost all patients, both the diabetic and the non-diabetic, were on β-blocker treatment (Table 1). Secondly, although the hemodynamic responses to dobutamine are reduced in diabetic patients [36] they normally still do respond. Consequently the non-responsiveness to β-adrenergic stimulation in the isolated right atrial cardiac muscles in our study should reside within the myocardium. Thirdly, although a reduced expression of β_1_-adrenoreceptors has been reported in myocardial tissue from diabetic patients undergoing CABG in the absence of β-blockers [37], to the best of our knowledge expression of β_1_-adrenoreceptors has never been reported in human myocardial tissue from diabetic patients with preserved EF. Interestingly, we did not observe a statistically significant reduction in β_1_-adrenoreceptors expression levels (Figure 3F) in the cardiac tissue from our diabetic cohort. Therefore, we suggest that the reduced β-adrenergic inotropic and lusitropic responses in the diabetic myocardium from patients with preserved EF are related to alterations in downstream β-adrenergic signaling pathways. Phosphorylation of PLB is an important downstream target of β-adrenergic signaling and we observed a markedly reduced PLB protein expression in the diabetic myocardium. Indirectly this implies there is less PLB to be phosphorylated and therefore this could be the reason for the reduced β-adrenergic inotropic and lusitropic responses in the diabetic myocardium from patients with preserved EF. Alternatively, Daniels *et al.* suggested that reduced β-adrenergic responsiveness in diabetes is related to metabolic changes leading to impaired energy conversion, which becomes apparent during physiological challenge [34]. Additionally, it was recently shown that although exercise limitations were similar between patients with reduced and preserved EF, significant differences occurred in their exercise-induced changes in systolic and diastolic properties, again reflecting their different underlying pathologies [38].

### Underlying mechanism of impaired relaxation

The underlying mechanism of the impaired relaxation may be related to impaired Ca^2+^ cycling within the cardiomyocytes. In human diabetic myocardium from patients with reduced EF a decreased level of RyR2 mRNA was observed [39]. In contrast, the human atrial myocardium from diabetic patients with preserved EF in our study showed no change in expression of the SR Ca^2+^ release channel RyR2, which is consistent with the preserved contractile function observed. Interestingly, in human non-diabetic and type 2 diabetic patients with preserved EF, it was reported that both SERCA2a and PLB expression were similar [26], and recently mRNA of SERCA was also shown to not be different between diabetic and non-diabetic spontaneously hypertensive rats [40], which is in contrast to our study showing an increased SERCA2a protein expression and a decrease in PLB protein expression. However, an altered mRNA level does not always equate to altered protein expression. Moreover, our observed changes in SERCA2a and PLB expression lead to an increase in the SERCA2a:PLB ratio in myocardial tissue of DM patients. From our data, which suggest accelerated SR Ca^2+^ uptake, it is tempting to speculate that the changes in protein density reflect a compensatory mechanism to attempt to normalize the impaired relaxation at rest. This is supported by the findings of Selby *et al.*[10] who suggested that in myocardial muscles obtained from non-diabetic patients with preserved EF, the impaired relaxation is accompanied by a disproportionate increase in SR Ca^2+^ content due to an increase in SR Ca^2+^ uptake. Notably, they found that increased SR Ca^2+^ content did not translate into a stronger contraction [10]. In addition, our results are supported by studies using rodent diabetic models that have reported an up-regulation of SERCA2a, a down-regulation of PLB and an improved contractile function following insulin treatment [19,41]. These authors showed that expression of SERCA2a mRNA in cardiomyocytes is under control of insulin, associated with changes in SR-Ca^2+^ uptake and phosphorylation of Akt, and inhibited by phosphoinositide 3 (PI3)-kinase inhibitor wortmanin [19]. This indicates that the underlying cellular mechanism for upregulation of SERCA2a could be mediated by the PI3-kinase-Akt-SERCA2a signaling cascade, suggesting that subtle changes in Ca^2+^ regulation, which promote diastolic dysfunction prior to overt systolic dysfunction, may be common to early stages of type 2 diabetes involving insulin resistance [19,42]. Moreover, it was shown recently that elevated oxidative stress may induce oxidative modifications of SERCA2a that could contribute to abnormal function in high-sucrose fed rats mimicking the metabolic syndrome heart [43].

In the present study, DM patients had an increase in hyperglycemic markers (glucose and HbA1_c_), despite all being on hyperglycemic control medication (metformin and insulin), and therefore changes in the expression of calcium-handling proteins might still be due to altered glycemic control. These changes in Ca^2+^ regulation may over time increase the energy demands of the diabetic myocardium and have important ramifications for future diabetic myocardium remodeling, potentially further impairing relaxation and eventually leading to myocardial dysfunction. Therefore, the increased SERCA2A:PLB expression ratio may be compensatory and beneficial for relaxation at resting conditions, but once the myocardium is challenged, there is little reserve for an increase in Ca^2+^ cycling leading to the lack of response to β-adrenergic stimulation observed in our study. Alternatively, in rodent models, experimental diabetes changed the phosphorylation of the sarcomeric protein troponin I [44,45] and also markedly shifted the myosin heavy chain (MHC) from the fast (V1) to the slow (V3) isoform [46,47], although the later might be less important in humans as most MHC already is predominantly in the slow isoform [48]. However, these studies indicate that myocardial relaxation can also be affected by alterations in regulatory myofilament proteins of the cardiac actomyosin system of which upon the effects of diabetes in humans are unknown.

A well-known underlying cause of diastolic dysfunction is increased myocardial fibrosis. The extracellular matrix remodeling plays an important role in cardiac fibrosis and the amount of extracellular collagen is caused by an imbalance between synthesis and degradation of collagen [49,50] and formation of advanced glycation end-products [51]. Cardiac fibrosis leads to increased myocardial stiffness, eventually resulting in both systolic and diastolic dysfunction [6]. Increased fibrosis is commonly observed in diabetic animal models (see review [50]), whereas reports in human cardiac tissue are conflicting [8,49]. In our study, the elevated collagen deposition in the right atrium of diabetic patients is indicative of increased fibrosis. Alternatively, increased diastolic myocardial stiffness could result from an increased cardiomyocyte stiffness, which relates to the elasticity of the giant cytoskeletal protein titin and is determined by its isoform expression [52] and/or posttranslational modification [53,54]. Changes in isoform expression of titin have been observed in type 1 diabetic animal models [47,55,56]. Recently in a metabolic animal model hyperphosphorylation of titin contributed importantly to underlying diastolic dysfunction [54]. However the specific role of titin regulating diastolic (dys) function, especially in type 2 diabetes in humans, is still not clear.

### Limitations

Due to the use and access to human tissue, there are a number of limitations to our study. CAD is known to be a co-morbidity and a specific causal factor for the transition from diastolic to systolic failure [22]. As all our patients had CAD for inclusion into the study, our data were not compared to healthy human myocardium, so it is unclear if our findings would translate to patients with DM but without CAD. Non-transplanted donor heart tissue is the closest available “non-diseased’ human myocardial tissue, and our force values obtained in right atrial cardiac muscles from non-DM patients with CAD and preserved EF were similar to data from LV myocardial tissue of non-transplanted donor hearts [28], although caution should still be taken that the function of the “non-diseased” hearts might still be affected by the cause of death (such as accident trauma), the use of cardioplegic solutions and the variable elapsed time between removal of the heart and the dissection of cardiac muscles. Nevertheless, diabetic patients with CAD are an extensive cohort [57] and are known to have double the risk of progressing to HF compared to non-diabetic CAD patients [58], and therefore our findings provide important novel and relevant knowledge to a clinically large and important patient group.

Another limitation is that differences in the ultrastructure and Ca^2+^ dynamics between the atria and ventricular myocardium exists [59] , which need to be carefully considered when translating right atrial findings to the left ventricular myocardium or the whole heart. For instance it has been shown that atrial tissue expresses relatively more SERCA2a and less PLB compared to ventricular tissue [60]. On the other hand, cardiac muscles obtained from right atrial appendages have been extensively used to study the function of human myocardium [9,26-28].

## Conclusions

In patients with CAD and preserved EF, the existence of type 2 diabetes resulted in impaired relaxation and increased fibrosis of the right atrium, with preserved myocardial contractile function. Our novel finding of increased expression of SERCA2a:PLB ratio suggests a compensatory mechanism to enhance relaxation and maintain the diastolic function at rest of the fibrotic diabetic atrial myocardium. We also demonstrate for the first time that human right atrial cardiac muscles from type 2 diabetic patients with preserved EF are unresponsive to β-adrenergic stimulation despite no change in expression of the β_1_-adrenoreceptor expression levels, suggesting impaired downstream β-adrenergic modulation and lack of contractile reserve. Our study provides novel knowledge on the underlying pathology of diastolic dysfunction in the diabetic myocardium.

## Abbreviations

A: Late ventricular filling velocity; BDM: 2,3-butanedione monoxime; BMI: Body mass index; BP: Blood pressure; CABG: Coronary artery bypass graft; CAD: Coronary artery disease; CSA: Cross sectional area; -dF/dtmin: Minimum rate of relaxation; +dF/dtmax: Maximum rate of contraction; Decel: Early ventricular deceleration time; DM: Type 2 diabetes mellitus; e’: Early ventricular myocardium relaxation velocity; E: Early ventricular filling velocity; EF: Ejection fraction; Fdev: Developed force; GAPDH: Glyceraldehyde 3-phosphate dehydrogenase; HbA1c: Glycated hemoglobin; IVSd: Interventricular septal diameter diastole; LA: Left atrial; LA-2D: Left atrial 2-dimensional diameter; Lmax: Muscle length at which isometric developed force was maximal; LV: Left ventricular; LVEDV: Left ventricular end diastolic volume; LVESV: Left ventricular end systolic volume; LVIDd: Left ventricular internal diameter diastole; LVIDs: Left ventricular internal diameter systole; LVPWd: Left ventricular posterior wall thickness end-diastole; MHC: Myosin heavy chain; non-DM: Non-diabetic; PBS: Phosphate buffered saline; PI3: Phosphoinositide 3; PLB: Phospholamban; PVDF: Polyvinylidene fluoride; RAA: Right atrial appendages; RT50: 50% relaxation time; RyR2: Ryanodine receptor 2; SDS: Sodium dodecyl sulphate; SERCA2a: Sarcoplasmic reticulum Ca^2+^ ATPase; SV: Stroke volume; Tau: Time constant of relaxation; TBD: Tris buffered saline; TtPF: Time to peak force.

## Competing interests

The authors declare that they have no competing interests.

## Authors’ contributions

SJL, HYW, IAEB and GH performed experimental work and primary analysis; RRL, SJL, and SC performed secondary analysis; RRL, AB, RK and PPJ managed experimental work; IFG, RWB, MJAW, PS, SC provided human tissue and patient data; RRL, SJL, and PPJ drafted the manuscript; AB, RK, JCB, PS, IFG, RWB, MJAW and SC edited manuscript; RRL and PPJ designed study. All authors read and approved the final manuscript.
